# Decoding HIV Discourse on Social Media: Large-Scale Analysis of 191,972 Tweets Using Machine Learning, Topic Modeling, and Temporal Analysis

**DOI:** 10.2196/76745

**Published:** 2025-08-29

**Authors:** Xiangming Zhan, Meijia Song, Cho Hee Shrader, Chad E Forbes, Angel B Algarin

**Affiliations:** 1 Edson College of Nursing and Health Innovation, Arizona State University, 500 N 3rd St, Phoenix, Phoenix, AZ, 85004, United States, 1 (330) 272-4294; 2 School of Nursing, University of Minnesota, Minneapolis, MN, United States; 3 Division of Nursing Science, School of Nursing, Rutgers University, Newark, NJ, United States

**Keywords:** HIV prevention, social media, topic modeling, temporal analysis, machine learning, public health informatics

## Abstract

**Background:**

HIV remains a global challenge, with stigma, financial constraints, and psychosocial barriers preventing people living with HIV from accessing health care services, driving them to seek information and support on social media. Despite the growing role of digital platforms in health communication, existing research often narrowly focuses on specific HIV-related topics rather than offering a broader landscape of thematic patterns. In addition, much of the existing research lacks large-scale analysis and predominantly predates COVID-19 and the platform’s transition to X (formerly known as Twitter), limiting our understanding of the comprehensive, dynamic, and postpandemic HIV-related discourse.

**Objective:**

This study aims to (1) observe the dominant themes in current HIV-related social media discourse, (2) explore similarities and differences between theory-driven (eg, literature-informed predetermined categories) and data-driven themes (eg, unsupervised Latent Dirichlet Allocation [LDA] without previous categorization), and (3) examine how emotional responses and temporal patterns influence the dissemination of HIV-related content.

**Methods:**

We analyzed 191,972 tweets collected between June 2023 and August 2024 using an integrated analytical framework. This approach combined: (1) supervised machine learning for text classification, (2) comparative topic modeling with both theory-driven and data-driven LDA to identify thematic patterns, (3) sentiment analysis using VADER (Valence Aware Dictionary and sEntiment Reasoner) and the NRC Emotion Lexicon to examine emotional dimensions, and (4) temporal trend analysis to track engagement patterns.

**Results:**

Theory-driven themes revealed that information and education content constituted the majority of HIV-related discourse (120,985/191,972, 63.02%), followed by opinions and commentary (23,863/191,972, 12.43%), and personal experiences and stories (19,672/191,972, 10.25%). The data-driven approach identified 8 distinct themes, some of which shared similarities with aspects from the theory-driven approach, while others were unique. Temporal analysis revealed 2 different engagement patterns: official awareness campaigns like World AIDS Day generated delayed peak engagement through top-down information sharing, while community-driven events like National HIV Testing Day showed immediate user engagement through peer-to-peer interactions.

**Conclusions:**

HIV-related social media discourse on X reflects the dominance of informational content, the emergence of prevention as a distinct thematic focus, and the varying effectiveness of different timing patterns in HIV-related messaging. These findings suggest that effective HIV communication strategies can integrate medical information with community perspectives, maintain balanced content focus, and strategically time messages to maximize engagement. These insights provide valuable guidance for developing digital outreach strategies that better connect healthcare services with vulnerable populations in the post–COVID-19 pandemic era.

## Introduction

HIV remains a significant global pandemic, with approximately 40 million people living with HIV and an estimated 1.3 million new diagnoses in 2023 [1]. In the United States, there are over 1.2 million people living with HIV, and more than 1 in 8 remain undiagnosed [2]. Access to information and health care services is crucial for HIV prevention and treatment, but is hindered by multiple barriers. At the individual level, stigma, financial constraints [3], fear, and psychological barriers often prevent people living with HIV from seeking medical services and engaging with the health care system [4]. As a result, individuals vulnerable to HIV often lack access to critical disease management information, leading many to turn to anonymous sources, such as digital platforms. At the institutional level, traditional clinic-based outreach models also struggle to effectively engage priority populations [5], such as youth, rural communities, and marginalized groups, which can widen disparities in HIV testing and viral suppression [6]. While digital platforms offer promising opportunities to reach individuals in their preferred internet-based environments**,** most health care providers lack evidence-based digital outreach strategies**,** a capability gap that has become increasingly evident in the post–COVID-19 era. This gap between health care delivery and digital engagement strategies undermines efforts to reach vulnerable populations, resulting in missed opportunities for HIV prevention, diagnosis, and treatment adherence, further perpetuating HIV transmission.

Social media platforms, including X (formerly Twitter), collectively process 456,000 posts per minute, generating an unprecedented volume of data transforming the spread of health information [7]. Because ordinary people’s voices resonate more deeply with audiences, community-driven information often receives more engagement than official health announcements [8]. As a result, grassroots influence on social media often shapes public understanding more powerfully than medical journals or traditional health campaigns [9]. For example, misinformation about HIV spreads far more rapidly than official information [10]. This shift presents a significant challenge to traditional health communication: despite health care institutions investing millions in health information distribution efforts, they struggle to match the speed and scale of information dissemination in digital spaces [11].

Social media’s role in HIV-related health communication has grown significantly. Studies have shown how these platforms serve as spaces for medical information sharing, personal narratives, and advocacy [12]. However, despite this growing body of work, important research gaps persist. First, most studies have narrowly focused on specific HIV-related topics like stigma reduction or pre-exposure prophylaxis (PrEP) use, rather than offering a broader landscape of HIV-related discourse [13-15]. Second, comprehensive analyses of large-scale public discussions—particularly those with datasets exceeding 100,000 tweets—remain rare [16,17]. Third, the majority of research predates COVID-19, which has influenced the circumstances and experiences of people living with HIV, creating a need for updated guidance [18,19]. Fourth, methodologically, existing HIV social media research employs either theory-driven approaches (eg, supervised classification based on established frameworks from previous literature) or data-driven methods (eg, unsupervised LDA) that allow themes to emerge naturally [20,21]. Each approach has limitations when used in isolation; theory-driven methods may miss emerging themes, while data-driven approaches may lack theoretical grounding. However, few studies have combined these complementary approaches, leaving significant knowledge gaps in understanding the comprehensive HIV-related discourse across social media platforms.

This study addresses these research gaps using an integrated analytical approach. We aimed to investigate current HIV-related topics and temporal trends on social media, providing insights for more effective public health communication strategies. To achieve this goal, our study uses a dual approach to topic modeling—combining data-driven and theory-driven methods—to capture both emergent themes and those grounded in established literature. This approach allows us to bridge the gap between theoretical frameworks and real-time social media discussions. Furthermore, by integrating sentiment analysis and temporal trend analysis, we provide a multidimensional view of HIV-related discourse, addressing both the emotional and practical aspects of these conversations.

Our study focuses on three core research objectives: (1) to observe the dominant themes in current HIV-related social media discourse; (2) to explore whether data-driven and theory-driven topic modeling approaches align with or diverge from each other; and (3) through sentiment analysis and temporal trend analysis, examine how emotional responses and temporal patterns influence the dissemination of HIV-related discourse on social media platforms.

## Methods

### Data Collection

Data for this study were collected through X’s application programming interface (API), a tool that allows developers to access and retrieve publicly available data from the platform [22]. We used the platform’s Basic tier access that permits retrieval of up to 10,000 tweets per month. In addition, the majority of our dataset consists of manually extracted public tweets collected over 5 months, which formed the primary source of our data. To ensure comprehensive coverage, 2 researchers conducted literature reviews and expert consultation methods [23]; the following keywords were used for inclusion: “LivingWithHIV,” “HIVPositive,” “HIVStories,” “HIVAware,” “EndHIVStigma,” “HIVDisclosure,” “HIVSupport,” “UequalsU,” “PrEP,” “AIDSAwareness,” “HIVAdvocate,” “HIVCommunity,” “HIVJourney,” “HIVTesting,” “FightHIV,” “HIVStigma,” “HIVCare,” and “HIVUndetectable.”

We collected tweets between June 1, 2023, and August 31, 2024, yielding an initial dataset of 229,340 tweets. The study team implemented comprehensive data preprocessing to optimize text quality. This process included: (1) text normalization through conversion to lowercase to ensure consistent term recognition [24]; (2) noise reduction by removing special characters, URLs, “@” mentions, hashtag symbols (while preserving the hashtag text content for thematic analysis), punctuation marks, and emojis to enhance topic modeling accuracy [25]; (3) linguistic filtering to exclude non-English character and non-ASCII (American Standard Code for Information Interchange) symbols to ensure compatibility with our algorithm; and (4) tokenization to segment text into individual terms for computational analysis [26]. In addition, we performed text normalization using lemmatization to reduce inflectional forms to their base forms (eg, “testing” to “test”), which helps consolidate semantically similar terms [27]. To address data redundancy, we applied deduplication algorithms that identified and removed identical and near-identical content, including retweets [28]. After completing all preprocessing steps, the final study sample consisted of 191,972 unique tweets, representing approximately 83.7% of the initial dataset.

### Ethical Considerations

This study involved secondary analysis of publicly available data and was granted exempt status through Arizona State University’s Institutional Review Board (IRB; STUDY00020345). To protect privacy, we removed all personally identifiable information during preprocessing. To address identifiability concerns, all user-generated content in our findings was either removed or paraphrased, ensuring that users cannot be traced through search engines. Since we analyzed only publicly available tweets, no informed consent was required. All data were stored in anonymized form on the research institution’s infrastructure to prevent unauthorized access. No compensation was provided to participants as this was an analysis of publicly available social media data.

### Data Analysis

#### Word Frequency Analysis

Using Python’s WordCloud library [29], we conducted a word frequency analysis to identify commonly used terms in HIV-related discourse. Our preprocessing involved a customized stopword filtering approach. Since our data collection used HIV-specific hashtags (LivingWithHIV, HIVPositive, EndHIVStigma, etc), the term “HIV” appeared ubiquitously across tweets, creating potential visualization bias. Through expert consultation, we determined that including “HIV” as a domain-specific stopword would enhance analytical clarity. In addition, we excluded standard English stopwords and generic discourse markers (“say,” “know,” “will”) to prevent skewed analysis. This preprocessing strategy enabled us to reveal meaningful, data-driven conceptual patterns beyond the omnipresent search terms.

#### LDA Topic Discovery

##### Theory-Driven Topic Discovery

Previous studies have explored the application of topic modeling in HIV research. Building upon the existing findings [14,18,19,30-34], we synthesized the semantic content of common topics and constructed 5 mutually exclusive categories of theoretical themes: (1) Information and education, encompassing medical information and public health communications; (2) Personal experiences and stories, including diagnosis narratives, treatment journeys, and disclosure accounts; (3) Opinions and commentary, comprising health care system feedback and policy perspectives; (4) Stigma and social impact, capturing discriminatory discourse and anti-stigma advocacy; and (5) Support and resources, focusing on support services, resource accessibility, and community networks (See details in Multimedia Appendix 1).

In our dataset, we use supervised machine learning to classify tweets into these 5 themes. A total of 2 researchers manually classified tweets (n=1000): Information and education (n=340), opinions and commentary (n=212), stigma and social impact (n=180), support and resources (n=177), and personal experiences and stories (n=91). To address class imbalance, we implemented synonym-based text augmentation (creating additional training examples by replacing words with their synonyms, while preserving meaning), expanding the dataset to 2427 samples [35].

To prepare the text data for classification, first, we used term frequency-inverse document frequency vectorization [26] to convert tweet text into numerical feature vectors; next, we divided the processed dataset into training (90%) and testing sets (10%); then, we compared logistic regression [36] and linear support vector machine [37] algorithms to determine which would provide better classification performance. For model optimization, we used grid search cross-validation to find optimal parameters (regularization parameter C=10) [38] and applied balanced class weights to handle data imbalance. Finally, we evaluated model performance using accuracy and macro-averaged *F*_1_-score metrics, with an acceptable threshold of *F*_1_-score≥0.75 for each category. After completing the tweet classification, we applied LDA [39] to each theme to establish more details, extracting 3 subtopics per theme to provide appropriate analytical granularity, resulting in a total of 15 topics.

##### Data-Driven Topic Discovery

Topic modeling is an unsupervised machine learning technique that generates a probabilistic model for a corpus of text data. The LDA topic modeling proposed by Blei et al [39] is a Bayesian mixture model, assuming that documents are randomly positioned over latent topics, each of which is categorized by a distribution over terms.

In our study, to explore topics without theoretical constraints, we also used a fully data-driven LDA approach. When performing LDA for topic modeling, it is necessary to manually set the number of topics and several critical parameters. Similar to Muhetaer and Hao’s research [40], in order to keep the accuracy of topic segmentation and subsequent topic extraction, we used GridSearch technology, combining it with measures through perplexity (which measures how well a model predicts unseen data, with lower values indicating better fit) and log-likelihood (which assesses how well the model fits the observed data, with higher values indicating better fit). By setting the range of topic numbers to “5-30” and learning rates to (0.5, 0.7, and 0.9), we conducted GridSearch and calculated the perplexity and log-likelihood values to determine the optimal number of topics and learning rate.

After the LDA model generated topic word distributions, we followed the guidelines of qualitative thematic analysis in computational text mining [41]; 2 researchers independently examined the top words for each topic, analyzing semantic patterns and word co-occurrence relationships to create descriptive labels. After the independent analysis, a third senior researcher was brought in to resolve any discrepancies and validate the preliminary labels. Once consensus was reached, the 3 researchers conducted label topic into broader themes based on their HIV expertise and formed the final themes through unanimous agreement.

### Sentiment Analysis

To understand emotional content in tweets, we conducted sentiment analysis using 2 well-known sentiment classifiers: the Valence Aware Dictionary and sEntiment Reasoner (VADER) and the National Research Council Canada Emotion Lexicon (NRC Emotion Lexicon) [42]. We selected both tools because VADER offers overall sentiment polarity optimized for social media language, while NRC offers granular emotional classification that VADER cannot capture. The VADER analyzer provided polarity scores (positive, negative, neutral, and compound—a unified score that combines the other 3 scores into a single sentiment metric ranging from −1 to +1) for each tweet. We then used the NRC Emotion Lexicon to map these tweets to 8 basic emotions (anger, fear, anticipation, trust, surprise, sadness, joy, and disgust) [43]. This mapping process integrated both the VADER sentiment scores and the presence of emotion-indicating terms from the NRC lexicon. The resulting emotion scores were minimum-maximum normalized to a 0‐1 scale to ensure comparability across the dataset.

### Trend Analysis

We used time-series visualizations to examine temporal patterns in HIV-related X engagement from June 2023 to August 2024. We analyzed both absolute metrics (total tweet volume) and normalized per-tweet engagement metrics (comments, transfers, favorites, bookmarks, and views) to account for varying posting frequencies. The visualizations were structured as multi-axis time-series plots to facilitate comparative analysis between related metrics while accommodating their different scales. We constructed four complementary visualizations: (1) average comments and transfers per tweet, (2) average favorites and bookmarks per tweet, (3) average views per tweet, and (4) monthly tweet volume.

## Results

### Visual Exploration of Word Frequency Analysis

Analysis of 191,972 tweets identified different term frequency patterns in HIV-related social media discourse. “Testing” and “support” emerged as the most prominent terms, reflecting the dual emphasis on clinical services and assistance in HIV conversations. Secondary high-frequency terms included “community” and “treatment.” Health-focused terms like “care” and “prevention” appeared with substantial frequency, while social impact terms such as “stigma” and “awareness” maintained a strong presence throughout the dataset. Action-oriented language including “fighting” and “need” consistently appeared, suggesting an advocacy dimension on the X platform.

### Theory-Driven LDA Analysis Results

Our theory-driven classification of HIV-related discourse revealed 5 distinct categories of content. The information and education theme represented the largest category (120,985/191,972, 63.02%), followed by opinions and commentary (23,863/191,972, 12.43%), personal experiences and stories (19,672/191,972, 10.25%), stigma and social impact (14,252/191,972, 7.42%), and support and resources (13,200/191,972, 6.88%). The supervised classification models achieved high performance metrics, with an accuracy of 0.91 and a macro *F*_1_-score of 0.90. Representative tweets for each category are presented in Table 1.

**Table 1.** Text classification results for HIV-related tweets (N=191,972).

Classification	n (%)	Representative tweets
Information and education	120,985 (63.02)	38M people live with HIV today, I want you to know. #PublicHealth
Opinions and commentary	23,863 (12.43)	Why aren’t we talking more about HIV prevention in rural communities? Urban areas get all the attention, but this affects everyone.
Personal experiences and stories	19,672 (10.25)	Living with HIV for 15 years now. With proper treatment and support, I’m healthier than ever. Don’t let fear stop you from getting tested.
Stigma and social impact	14,252 (7.42)	It’s 2024 and some people still believe you can’t share utensils or hug someone with HIV. This misinformation creates barriers in families, workplaces, and communities. Let’s fight stigma with education and compassion. #HIVAwareness
Support and resources	13,200 (6.88)	Free HIV testing and counseling services available at our community health center.

Following our theory-driven LDA approach, we further analyzed each of the 5 predefined theoretical themes to extract 3 subtopics within each theme. This resulted in 15 total subtopics that provided additional granularity to our predetermined theoretical framework. Each theme—information and education, personal experiences and stories, opinions and commentary, stigma and social impact, and support and resources—contained 3 subtopics with distinctive top word distributions as presented in Table 2.

**Table 2.** Theory-driven Latent Dirichlet Allocation (LDA) topics in HIV-related tweets on X.

Theme		Top words
Information and education		
Africa		million, article, africa, south_africa, population, adults, research, black, according, age
Youth health care		health, research, discrimination, program, prevention, healthcare, challenges, youth, adolescents, plhiv
Prevention		risk, antiretroviral, undetectable, individuals, arv, prevent, pregnant, transmission, effective, healthy
Personal experiences and stories		
Living with HIV		undetectable, community, black, healthy, thami_openly, asked, later, proud, diagnosis, medical
Survival stories		health, journey, research, home, needs, history, able, survivor, stone, start
Personal feelings		school, loved, words, heart, boy, far, morning, hate, cancer, given
Opinions and commentary		
Medical perspectives		undetectable, health, cure, spread, cancer, folks, medical, risk, safe, partner
Social issues		healthy, wrong, needs, poor, trans, black, normal, called, crime, loved
Government policy		vaccine, imagine, fauci, start, arv, government, diseases, healthcare, transmission, issue
Stigma and social impact		
Racial		health, discrimination, south_africa, black, africa, south_africans, healthy, nigerians, leave, start
Community stigma		undetectable, community, y’all, government, million, fear, wrong, lgbtq, cancer, eating
Interpersonal impact		partner, job, infections, wizarab, normal, parents, lie, society, absolutely, dying
Support and resources		
Community support		register, campaign, lgbtq, celebrate, community, group, housing, black, supporting, service
Health resources		health, healthy, information, safe, info, message, stand, spread, resources, hivawareness
Mental health support		health, discrimination, individuals, prevention, mental_health, needs, research, quality, adolescents, impact

### Data-Driven LDA Analysis Results

Based on our data-driven LDA topic model analysis, we identified 15 as the optimal number of topics. As shown in Table 3, perplexity decreased from the topic number 5 to 15 and then increased for higher number of topics. Similarly, log-likelihood reached its peak at 15 topics. GridSearch determined that a learning rate of 0.9 provides the best performance for this configuration, where both evaluation metrics achieve their optimal values simultaneously. This parameter selection represents the optimal tradeoff between model complexity and interpretability. Fewer topics result in oversimplified representations that miss important thematic nuances, while more topics lead to oversegmentation that reduces interpretative clarity. See Multimedia Appendix 2 for visualization.

**Table 3.** Perplexity and log-likelihood for different topic numbers in data-driven Latent Dirichlet Allocation (LDA) model.

Topic numbers	Perplexity	Log-likelihood
5	1348.43	−12,960,752.33
10	1323.48	−12,927,162.41
15	1294.01	−12,886,673.25
20	1334.87	−12,942,570.19
25	1349.78	−12,962,557.90
30	1392.94	−13,019,154.21

After analyzing the top word distributions, 3 researchers constructed topic labels and reached consensus. They identified patterns of thematic similarity and finally organized the topics into 8 broader thematic themes: community experiences, medical and epidemiological information, global perspectives, social commentary, stigma and discrimination, support and services, policy and governance, and prevention (see Table 4).

**Table 4.** Data-driven Latent Dirichlet Allocation (LDA) topics in HIV-related tweets on X (formerly Twitter).

Theme		Top words
Community experiences		
LGBTQ+^a^ experiences and advocacy		national, awareness, gay, spread, disease, journey, infected, gender, education, break medical
Living with HIV		treatment, undetectable, visit, life, healthy, drug, effective, medication, partners, information
People of color’s experiences and advocacy		black, sexual, raise, safe, sexual health, raise awareness, family, lgbtq, rights, service
Medical and epidemiological information		
HIV medical impact		medical, children, home, results, death, number, STIs, diagnosed, advocacy, population
Epidemic response and screening		fight, public, epidemic, screening, hepatitis, cases, state, ending, public health, infections
Global perspectives		
Global HIV context		health, africa, global, study, south, needs, resources, center, kits, change
Social commentary		
Digital social commentary		COVID, youth, project, read, raising, social, white, people, media, raising awareness
Stigma and discrimination		
Anti-stigma campaigns		stigma, support, fighting stigma, program, important, girls, barriers, school, progress, diagnosis
Discrimination awareness and action		status, stop, discrimination, campaign, response, individuals, lead, aware, positive, antiretroviral
Support and services		
Access to care services		services, blood, STI, confidential, efforts, improve, quality, available, tomorrow, register
Treatment innovation and cure		patients, vaccine, providing, system, patient, cure, difference, primary, vital, immune
Policy and governance		
Policy and funding initiatives		cancer, hope, funding, data, months, department, policy, happy, shows, levels
Prevention		
General prevention strategies		prevention, transmission, prevention treatment, condoms, control, key, government, risk, communities, article
PrEP^b^ and biomedical prevention		prep, sex, research, million, protect, child, ensure, power, watch, pep
Community-led prevention		community, infection, impact, advocate, exposure, prophylaxis, options, promote, exposure prophylaxis, meeting

a LGBTQ+: lesbian, gay, bisexual, transgender, queer, and other sexual and gender minority.

b PrEP: pre-exposure prophylaxis.

### Sentiment Analysis Results

**Figure 1.**
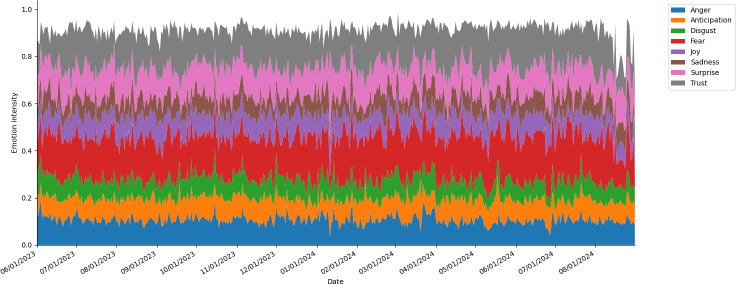
Temporal distribution of 8 emotions in HIV-related tweets.

The sentiment analysis showed the temporal distribution of the 8 emotions across our dataset from June 2023 to August 2024 (see Figure 1). As shown in the stacked area chart, where the y-axis represents normalized emotional intensity, fear, anger, and trust appear as dominant emotions throughout the study period. While the overall distribution of emotions remained relatively stable, temporal analysis demonstrated subtle fluctuations in emotional intensity, particularly during public health announcements and awareness activities.

### Trend Analysis Results

**Figure 2.**
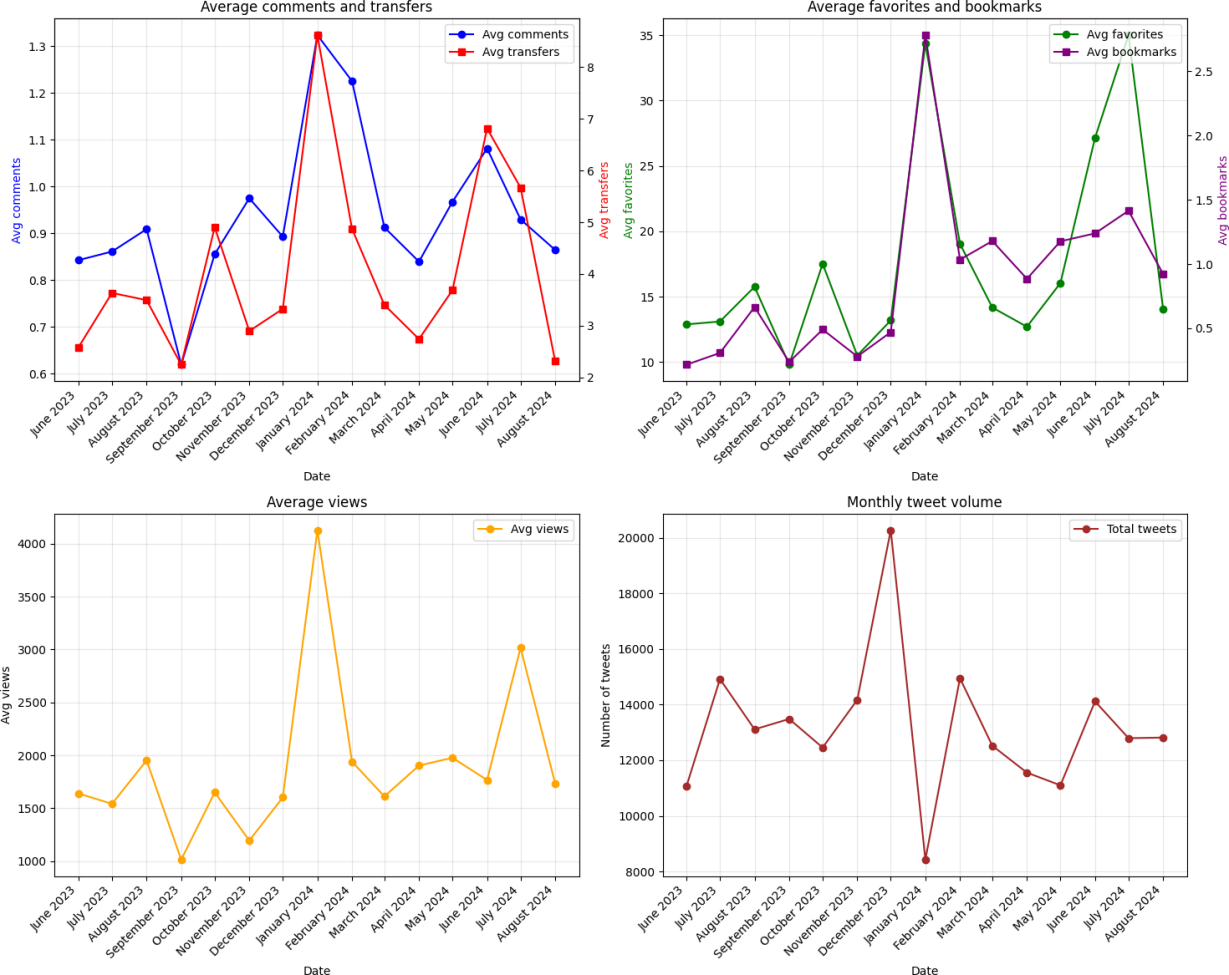
Monthly engagement metrics trend in HIV-related tweets. Avg: average.

Our analysis of tweet engagement metrics (n=191,972) revealed distinct temporal patterns around HIV awareness events (see Figure 2). The tweet volume reached its highest point during World AIDS Day in December 2023 (approximately 20,000 tweets), with subsequent peak engagement metrics in January 2024, including comments (1.3 per tweet), transfers (8 per tweet), and views (4000 per tweet). A second period of high activity occurred during National HIV Testing Day and Zero HIV Stigma Day in June-July 2024, when both posting volume (14,000 tweets) and engagement metrics increased simultaneously, with views reaching 3000 per tweet and favorites peaking at 32 per tweet.

## Discussion

### Principal Findings

This study presents a comprehensive analysis of HIV-related discourse on 191,972 tweets over 14 months from June 2023 to August 2024. Our primary contribution is the innovative combination of theory- and data-driven LDA approaches, offering complementary perspectives on HIV discourse that neither method could achieve alone. Our approach integrated 4 complementary methods: artificial intelligence methods for text classification and topic modeling, qualitative thematic analysis for contextual interpretation, sentiment analysis for emotional content understanding, and traditional observational methods for temporal trend analysis. Our dual theory and data-driven topic modeling approaches help bridge the gap between theoretical frameworks and real-world public discourse, revealing both established knowledge and emerging public concerns in HIV communication.

### Overview of Theory-Driven LDA Findings

The theory-driven LDA approach organizes the discourse within 5 comprehensive themes: information and education, personal experiences and stories, opinions and commentary, stigma and social impact, and support and resources. We further developed these themes into 15 subtopics to allow for more detailed analyses. These categories are shaped by previous theoretical framework expectations and reflect academic concerns with structured, future-oriented responses to the HIV epidemic. This process not only strengthens the existing theoretical framework but also supports their continuity and evolution in academic research [44]. For instance, the prominence of youth health care and prevention programs may reveal an investment in long-term health education and intervention. Similarly, discussions about government policy and medical ethics may mirror the scholarly emphasis on institutional responsibility and systemic solutions [45].

In this sense, the theory-driven approach plays a critical role in reinforcing, elaborating, and updating existing frameworks. The “Information and education” theme includes subtopics like health care in African contexts, youth-specific prevention, and public health messaging. “Personal experiences and stories” offers a more emotional layer, capturing the realities of living with HIV, survival narratives, and personal reflections. “Opinions and commentary” points to structured discussions around medical perspectives, social issues, and government policies. The themes of “Stigma and social impact” and “Support and resources” cover interpersonal and racial stigma, as well as community-based and mental health support. Altogether, the 15 subcategories reflect a well-rounded academic framing that integrates institutional, medical, emotional, and social dimensions of the HIV conversation.

### Overview of Data-Driven LDA Findings

The data-driven LDA method captured 15 topics that emerged organically from public discussions, which we further organized into 8 broader themes: Community experiences, medical and epidemiological information, global perspectives, social commentary, stigma and discrimination, support and services, policy and governance, and prevention. While it may lack the clear structure of theory-led models, it uncovered discursive themes that are overlooked in research. Examples include digital commentary on the intersection of HIV and COVID-19, grassroots prevention campaigns, and anxieties around funding and service provision.

In this sense, the data-driven approach reveals what matters most to users in the real world on discussion platforms, from LGBTQ+ and people of color advocacy to personal treatment narratives and critiques of health care access. Community experiences include LGBTQ+advocacy, personal treatment experiences, and POC-led discussions, while medical and epidemiological information focuses on disease impact, diagnosis statistics, and public screening efforts. Global perspectives reintroduce the African context but with a broader emphasis on global disparities. Social commentary captures how HIV is discussed alongside other major issues like COVID-19, particularly through digital media. The prevention cluster extends from traditional messaging to community-led initiatives and biomedical approaches like PrEP [46]. Overall, these themes reveal a bottom-up perspective, highlighting lived experience, advocacy, and the urgency of practical solutions—dimensions that are often underrepresented in traditional academic models.

### Points of Convergence: Shared Focus Areas

Despite different methodological approaches, both theory- and data-driven LDAs converge on several key themes, highlighting a shared prioritization across theory and discourse. (1) Prevention is a critical area in both analyses, with the theory-driven model focusing on biomedical messaging (eg, antiretroviral use), and the data-driven model elaborating on multiple approaches, including PrEP and community-led efforts. This suggests that prevention-centered interventions should integrate both biomedical messaging (eg, antiretroviral use) from theory-driven approaches with the community-led and PrEP-focused strategies highlighted in data-driven analysis. (2) Lived Experiences with HIV are central across both models. The theoretical model dissects structured narratives (eg, survival stories), while the data-driven results foreground advocacy (eg, LGBTQ+ experiences) and treatment-sharing communities [47]. (3) Stigma is addressed comprehensively: racial, community, and interpersonal impact in the theory-driven model parallel anti-stigma campaigns and discrimination awareness in the data-driven analysis [48]. It points to intervention needs that address racial, community, and interpersonal dimensions through coordinated antistigma campaigns. (4) Support systems, though framed differently, appear in both. The theory-driven approach emphasizes community and mental health support, while the data-driven model foregrounds access issues, service delivery, and biomedical innovation [49]. It suggests that interventions should bridge institutional mental health resources with community-based service delivery innovations for comprehensive care. (5) Global health and Africa feature prominently in both, underscoring shared recognition of HIV’s global dimensions—though the data-driven themes offer a more explicitly labeled Global HIV Context.

These areas of convergence not only validate the robustness of certain themes across both analytical approaches, but also illustrate how the 2 mindsets behind them—top-down and bottom-up—can complement each other and be integrated in public health discourse analysis.

### Key Differences and Their Significance

The most important divergence lies in what each approach reveals that the other does not. The data-driven approach identifies 3 themes absent from the theory-guided structure: Digital social commentary, policy and funding initiatives, and community-led prevention. These themes reflect real-time societal reactions, participatory politics, and community-driven change—critical aspects often missing in literature-based modeling [50]. In contrast, the theory-driven model includes nuanced psychosocial categories like personal feelings or medical perspectives that reflect deeper affective or clinical framing not prominent in spontaneous X discourse.

These differences are not merely methodological—they highlight actionable gaps between public discourse and academic frameworks. Data-driven themes capture lived urgency and sociopolitical energy, while theory-driven themes offer structure, historical continuity, and future-oriented [51]. Effective HIV communication requires bridging this gap by integrating grassroots narratives with evidence-based frameworks to ensure messages resonate with target communities while maintaining scientific accuracy [52].

### Word Cloud Findings

The word cloud analysis reveals “testing” and “support” as dominant terms in HIV-related social media discourse, reflecting emphasis on both clinical services and community assistance. Secondary terms like “treatment,” “care,” “prevention,” and “community” demonstrate the focus on health interventions [53], while terms like “stigma” and “awareness” highlight social dimensions [54]. The relative absence of policy-related terminology suggests public discourse may emphasize individual experiences over structural solutions, indicating an opportunity for health communicators to expand discussions around systemic approaches to HIV response [55].

### Sentiment Analysis Findings

Sentiment analysis of HIV-related discourse revealed a complex emotional landscape where trust appears alongside negative emotions like fear and sadness throughout the study period. While emotional distribution remained relatively stable over time, subtle fluctuations occurred during health announcements and awareness campaigns. These findings suggest that HIV discourse encompasses both concern (represented by fear and sadness) and confidence (represented by trust), likely reflecting public responses to risk information alongside messages about treatment advances and support resources [56]. The persistent presence of trust may indicate growing public confidence in HIV interventions, while the concurrent fear highlights ongoing concerns about transmission and stigma [57]. This emotional duality underscores the importance of balanced communication strategies that acknowledge concerns and fears while reinforcing efficacy and support messages. This strategy—pairing the experience of fear with solutions that can help alleviate the fear—has been established as a highly effective persuasion technique in public health campaigns for decades [58].

### Temporal Analysis Findings

Interestingly, consistent with a multitude of past research specific to social media [59,60], emotional fluctuations often coincided with periods of heightened social media activity around major awareness events, suggesting a possible link between emotional dynamics and patterns of public engagement. Temporal analysis revealed 2 distinct patterns of social media interaction around major HIV awareness events: a delayed engagement pattern following institutional communications and an immediate engagement pattern driven by community-driven messages. The first pattern emerged during World AIDS Day in December 2023, where a sequential engagement emerged; in this case, peak tweet volume (20,000 tweets) occurred before peak engagement metrics in January 2024. This delayed pattern may reflect the nature of peer influence in institutional health communication: official agencies and health organizations initially disseminated formal health messages and campaign information, creating an information-rich environment that requires time for peer networks to process. The lag in engagement illustrates how individuals process health information in stages: first absorbing official messages, then contextualizing them locally, and finally transforming them into relevant peer-to-peer discussions [61]. In contrast, the second pattern during National HIV Testing Day and Zero HIV Stigma Day in June-July 2024 showed simultaneous increases in both tweet postings and engagement. This surge coincided with high participation from HIV-affected communities, including people living with HIV, their support networks, health care providers, and advocacy groups. When members of these interconnected communities share personal experiences related to testing or stigma, they strengthen existing social bonds and prompt immediate engagement from others who share similar experiences or support roles [62]. These contrasting patterns highlight how peer influence operates differently in different networks: institutional communications lead to gradual, top-down information sharing, while community-based messages trigger immediate peer engagement through horizontal social network connections [63].

### Implications for Practice, Policy, and Research

For health care practitioners, our findings reveal that HIV discourse contains a complex emotional blend of trust, fear, and sadness. This suggests that, consistent with other public health campaigns [58], communication strategies should acknowledge these diverse emotions. Clinicians should integrate supportive messaging that addresses emotions while providing factual information. Our temporal analysis identifies strategic timing opportunities during awareness events when both posting volume and user interaction peak. Policymakers can use these insights to design interventions that address the disconnect we identified between individual experiences and structural solutions [64], particularly in addressing stigma themes that appeared prominently in both analytical approaches. Future research should build on our multimethod framework to further explore underrepresented topics in HIV social media discourse, examine how digital platforms continue to shape public health communication, and leverage these data with key behavioral outcomes like attendance at local health events, clinic visits, and engagement with public health resources [65].

### Study Limitations

This study has several limitations. First, data collection was limited to the X platform, which may not represent the full spectrum of social media HIV discourse. Second, our 14-month analysis period captures only recent trends, potentially missing longer-term patterns in HIV-related communication. Third, our sentiment analysis was conducted at the overall dataset level rather than within specific topics, which limits our understanding of how emotional response may vary across different topics. Fourth, our analysis focused solely on text content without incorporating visual media such as images and videos, which could provide additional context. While our topic modeling approach using LDA is appropriate for text data, future research would benefit from multimodal analysis that integrates both textual and visual elements for a more comprehensive understanding of HIV communication on social media.

### Conclusions

This study demonstrated the value of a multimethod approach in analyzing HIV-related social media discourse. Our parallel analysis through theory-driven and data-driven LDA revealed complementary perspectives on HIV communication on social media. Word frequency analysis identified “testing” and “support” as dominant themes, while sentiment analysis showed a complex emotional landscape with trust appearing prominently alongside fear and sadness throughout the study period. Temporal analysis demonstrated strategic opportunities during HIV awareness events when both posting volume and engagement metrics peak significantly. These findings underscore the importance of developing HIV messaging that integrates medical information with community perspectives, addresses the emotional complexity revealed in our sentiment analysis, and leverages the timing patterns identified in our temporal data to maximize reach and impact. As social media platforms increasingly serve as accessible sources of information when traditional health resources may present barriers, these communication strategies become even more essential for effective public health outreach.

## Supplementary material

10.2196/76745 Multimedia Appendix 1 Theoretical framework (Latent Dirichlet Allocation).

10.2196/76745 Multimedia Appendix 2 Detailed learning rate in data-driven Latent Dirichlet Allocation (LDA) model.

## Abbreviations

API: application programming interface
LDA: Latent Dirichlet Allocation
PrEP: pre-exposure prophylaxis
VADER: Valence Aware Dictionary and Sentiment Reasoner
