# Supplementary material for: Decoding HIV Discourse on Social Media: Large-Scale Analysis of 191,972 Tweets Using Machine Learning, Topic Modeling, and Temporal Analysis
Source: J Med Internet Res. 2025 Aug 29;27:e76745. doi: 10.2196/76745 (PMC12396797; doi:10.2196/76745)
Supplement: Multimedia Appendix 1 [file jmir-v27-e76745-s001.docx]

1. Theoretical Framework Development

We applied deductive thematic synthesis to previous HIV/AIDS social media studies (Table 1). This approach enabled us to develop five thematic categories that comprehensively capture HIV/AIDS discourse on social media: (a) Information & Education; (b) Personal Experiences & Stories; (c) Opinions & Commentary; (d) Stigma & Social Impact; and (e) Support & Resources. This classification approach aligns with established frameworks in public health discourse analysis for analyzing vulnerable population discourse. To ensure reliability, two researchers independently coded the data, then reached consensus through comparative analysis and discussion. This systematic approach enhanced the rigor and credibility of our thematic categorization results.

Table S1.Summary of Previous Topic Modeling Studies on HIV/AIDS-Related Social Media Content (2018-2024)

| **Study** | **Data Source** | | **Sample Size** | **Identified Topics** |
| --- | --- | --- | --- | --- |
| Lohmann et al.(2018) | | Twitter | 1,000 | Informative contents, behavior change strategies advoncy, jokes or insults. |
| Liu & Lu (2018) | | Baidu Tieba | 76,865 | HIV/AIDS testing, treatment, and HIV-related consultation |
| Lomotey et al. (2023) | | Twitter | 2,839,091 | Sexuality and Lifestyle Choices of PLWHA, Testing for HIV, HIV/AIDS Prevention, Healthcare and Support Resources, Stigmatization, HIV impact on minority groups such as Women and Children, the Role of Government, Related Diseases to HIV, HIV Criminalization and Disclosure, Research on HIV, Misinformation on HIV, Outbreak of HIV, HIV Treatment, COVID-19 syndemic. |
| Malik et al. (2021) | | Twitter | 160,658 | events and activities information, preventive measures, treatment options, stigma, and call for additional resources. |
| Stevens et al.(2020) | | Twitter | 2157 | HIV-prevention |
| Burgess et al.(2022) | | Twitter | 69,197 | prevention |
| Wang et al.(2024) | | Twitter | 6,439 | stigmatizing jokes and insults; awareness, stigma, HIV criminalization,violence, LGBTQIA + , and women’s rights, risk and wellness information |
| Odlum et al.(2018) | | Twitter | 118, 155 | Prevention, Support, Treatment or Care |

References:

1. Lohmann S, Lourentzou I, Zhai C, Albarracín D. Who is Saying What on Twitter: An Analysis of Messages with References to HIV and HIV Risk Behavior. Acta Investig Psicol. Apr 2018;8(1):95-100.
2. Liu C, Lu X. Analyzing hidden populations online: topic, emotion, and social network of HIV-related users in the largest Chinese online community. BMC Med Inform Decis Mak. Jan 5, 2018;18(1):2.
3. Malik A, Antonino A, Khan ML, Nieminen M. Characterizing HIV discussions and engagement on Twitter. Health Technol. Nov 2021;11(6):1237-1245.
4. Stevens R, Bonett S, Bannon J, et al. Association Between HIV-Related Tweets and HIV Incidence in the United States: Infodemiology Study. J Med Internet Res. Jun 24, 2020;22(6):e17196.
5. Burgess R, Feliciano JT, Lizbinski L, Ransome Y. Trends and Characteristics of #HIVPrevention Tweets Posted Between 2014 and 2019: Retrospective Infodemiology Study. JMIR Public Health Surveill. Aug 11, 2022;8(8):e35937.
6. Wang Y, Bannon JA, Roszkowska N, et al. From virus to viral: content analysis of HIV-related Twitter messages among young men in the U.S. BMC Digit Health. Jul 30, 2024;2(1):44.
7. Odlum M, Yoon S, Broadwell P, Brewer R, Kuang D. How Twitter Can Support the HIV/AIDS Response to Achieve the 2030 Eradication Goal: In-Depth Thematic Analysis of World AIDS Day Tweets. JMIR Public Health Surveill. Nov 22, 2018;4(4):e10262.
