# Supplementary figures and images for: Decoding HIV Discourse on Social Media: Large-Scale Analysis of 191,972 Tweets Using Machine Learning, Topic Modeling, and Temporal Analysis
Source: J Med Internet Res. 2025 Aug 29;27:e76745. doi: 10.2196/76745 (PMC12396797; doi:10.2196/76745)

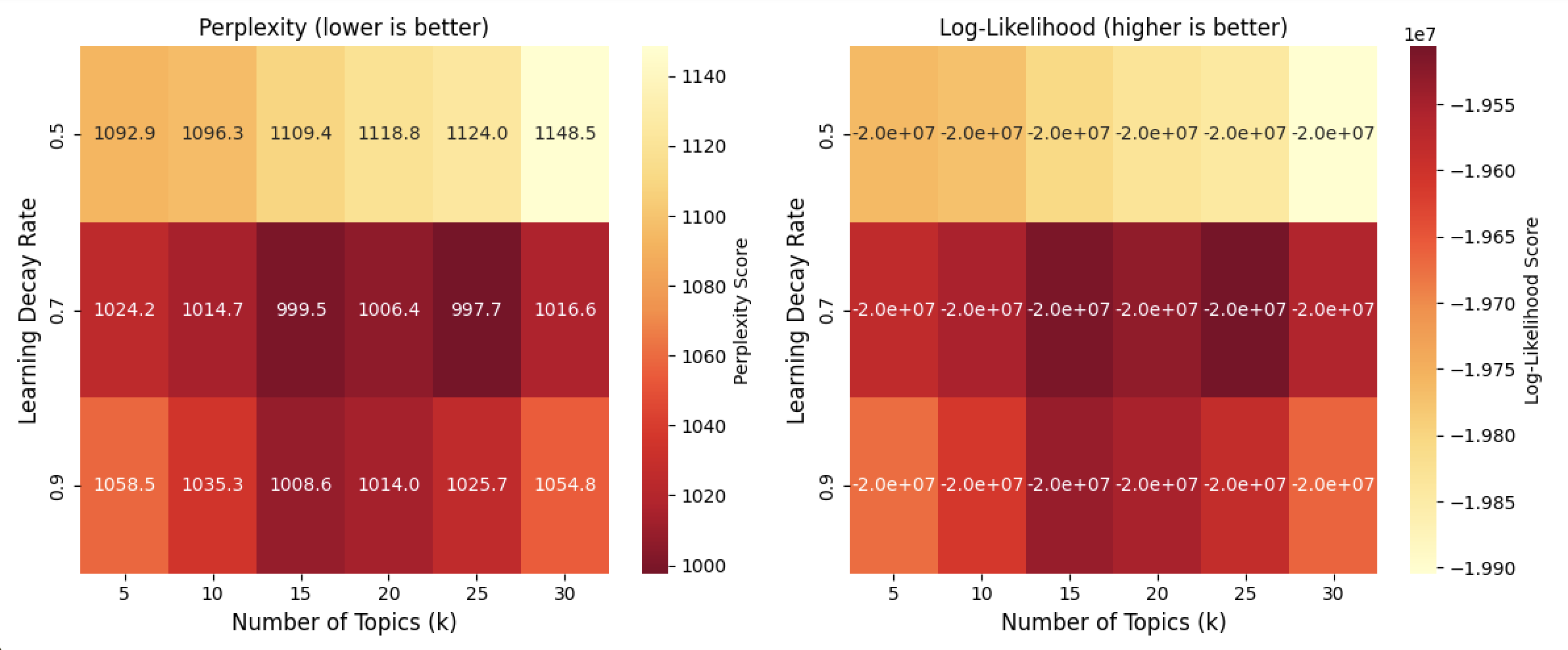

Supplement: Multimedia Appendix 2 [file jmir-v27-e76745-s002.png]
